# P-150. Increased detection of Shiga-toxin producing Escherichia coli in adults’ stool specimens with multiplex infectious diarrhea panel nucleic-acid amplification testing

**DOI:** 10.1093/ofid/ofaf695.376

**Published:** 2026-01-11

**Authors:** Eugene Yeung

**Affiliations:** University of British Columbia

## Abstract

**Background:**

Shiga-toxin producing *Escherichia coli* (STEC) infection is a cause of travelers’ diarrhea. Since 2022, diagnostic laboratories in British Columbia (BC), Canada, have been advised to replace traditional bacterial stool culture testing with multiplex infectious diarrhea panel nucleic-acid amplification testing (IDP-NAAT), which contains a minimum of 14 common pathogens, including STEC, which is known to cause hemolytic uremia syndrome with antimicrobial use. NAAT is generally more sensitive than culture testing. The current retrospective audit investigated whether multiplex IDP-NAAT would lead to better detection of STEC in adult patients.

**Methods:**

LifeLabs BC microbiology laboratories, connected with 129 collection centres in urban and rural communities in the province, provided the laboratory data on STEC results from adult patients (age >18years). An audit was conducted from September 2022 to August 2023, one year prior to implementation of IDP-NAAT in LifeLabs BC, when traditional culture plates were used to grow STEC with selective sorbitol MacConkey agar media, followed with NAAT. Another audit was conducted from October 2023 to September 2024, when IDP-NAAT was the primary method to detect STEC in primary stool specimens. Chi-square with Yates correction was used to compare the change.

**Results:**

Prior to implementation of IDP-NAAT, 22 of 3897 adult patients’ stool specimens (0.056%) was positive for STEC. After the implementation of IDP-NAAT, 522 of 55170 of adult patients’ stool specimens was positive for STEC (0.946%; *p* < 0.05 vs. prior).

**Conclusion:**

The positivity rate and number of STEC positive results among adult patients’ stool specimens were significantly higher after the implementation of IDP-NAAT. The results are suggestive of a pseudo-outbreak due to change in test methodology. More communications and educations may be warranted to remind clinicians that antimicrobial use in STEC infection does not improve symptoms but can cause hemolytic uremia syndrome.

**Disclosures:**

All Authors: No reported disclosures

